# National Liver Cancer Screening Trial (TRACER) study protocol

**DOI:** 10.1097/HC9.0000000000000565

**Published:** 2024-11-04

**Authors:** Amit G. Singal, Neehar D. Parikh, Fasiha Kanwal, Jorge A. Marrero, Sneha Deodhar, Stephanie Page-Lester, Camden Lopez, Ziding Feng, Nabihah Tayob

**Affiliations:** 1 Department of Internal Medicine, UT Southwestern Medical Center, Dallas, Texas, USA; 2 Department of Internal Medicine, University of Michigan, Ann Arbor, Michigan, USA; 3 Department of Medicine, Baylor College of Medicine, Houston, Texas, USA; 4 Department of Internal Medicine, University of Pennsylvania, Philadelphia, Pennsylvania, USA; 5 Biostatistics Program, Public Health Science Division, Fred Hutchinson Cancer Research Center, Seattle, Washington, USA; 6 Department of Data Science, Dana Farber Cancer Institute, Boston, Massachusetts, USA

**Keywords:** biomarker, GALAD, liver cancer, screening, ultrasound

## Abstract

**Background:**

Professional guidelines recommend HCC screening in at-risk patients using semi-annual ultrasound with or without alpha-fetoprotein (AFP); however, this strategy has limited effectiveness due to low adherence and sensitivity. Increasing data support the potential role of blood-based biomarker panels, which could improve both aspects. The biomarker panel GALAD, comprised of sex, age, and 3 blood biomarkers (AFP, AFP-L3, and des-carboxy prothrombin des-carboxy prothrombin), has shown high sensitivity and specificity in biomarker phase II (case-control) and phase III (retrospective cohort) validation studies. However, prospective validation in a large phase IV biomarker clinical utility trial is necessary before its adoption in practice.

**Methods:**

The National Liver Cancer Screening Trial is an adaptive pragmatic randomized phase IV trial, which began enrollment in January 2024, comparing ultrasound-based versus biomarker-based screening in 5500 patients with chronic hepatitis B infection or cirrhosis from any etiology. Eligible patients are randomly assigned in a 1:1 ratio to semi-annual screening with ultrasound ± alpha-fetoprotein (arm A) or semi-annual screening with GALAD (arm B). Randomization is stratified by enrollment site, liver disease severity (per Child-Pugh class), liver disease etiology (viral, nonviral, and noncirrhotic HBV), and sex. Patients are being recruited from 15 sites (a mix of tertiary care academic referral centers, safety-net health systems, and large community health systems) over a 3-year period, and the primary endpoint, reduction in late-stage HCC, will be assessed at the end of year 5.5.

**Discussion:**

The results of this trial will inform the best strategy for HCC screening and early-stage detection in patients with chronic liver diseases. If GALAD shows superiority, HCC screening would primarily shift from an ultrasound-based strategy to the adoption of the biomarker panel.

**Trial Registration:**

NCT06084234.

**Trial Status:**

The TRACER Study is actively enrolling.

## Graphical abstract

**Figure:**
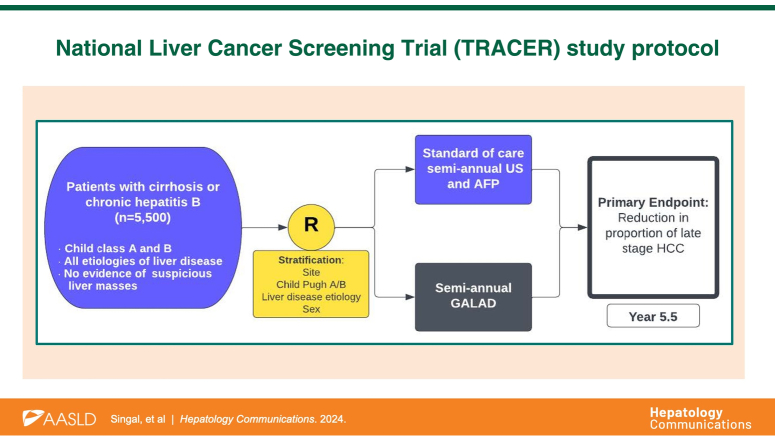

## INTRODUCTION

### Background and rationale

HCC is the fourth leading cause of cancer-related death worldwide and one of the few cancers with rising mortality in the Western world.^1^ HCC primarily occurs in patients with cirrhosis and chronic hepatitis B (HBV) infection and is the leading cause of cancer-related death in these populations. With the increased use of antiviral therapies, there has been a shift in etiologic risk factors from HBV and hepatitis C infection to an increasing proportion of cases due to alcohol-associated liver disease and metabolic dysfunction–associated steatotic liver disease.^2^^,^^3^ The risk of HCC can significantly vary in patients with cirrhosis and HBV infection, with patient age, sex, race and ethnicity, liver disease etiology, and cirrhosis severity being well-recognized risk factors.^4^^–^^6^

HCC prognosis is strongly driven by tumor burden, degree of liver dysfunction, and patient performance status.^7^ Curative treatment options yield a median 5-year survival exceeding 70% in patients with early-stage HCC, whereas those with late-stage HCC primarily undergo palliative therapies and have a 5-year survival below 20%.^8^ Despite professional society guidelines recommending HCC screening in at-risk patients,^9^^–^^11^ a minority of patients with HCC are detected at an early stage. These poor outcomes are largely due to limitations with the current strategy for screening and early detection of HCC in at-risk patients.

Ultrasound with or without alpha-fetoprotein (AFP) has been the cornerstone of HCC screening for over 2 decades, with the American Association for the Study of Liver Disease (AASLD) recommending the combination, ultrasound plus AFP, and the European Association for the Study of the Liver (EASL) recommending ultrasound alone. This strategy is supported by a large randomized controlled trial (RCT) in patients with chronic HBV infection, demonstrating improved early-stage HCC detection and improved HCC-related survival in the ultrasound arm,^12^ although it is unclear if these results apply to patients with cirrhosis given increased liver nodularity potentially impacting ultrasound sensitivity. Data from cohort studies in patients with cirrhosis suggest suboptimal accuracy of ultrasound-based screening for early-stage HCC detection, highlighting a need for improved screening modalities.^13^ A meta-analysis reported a sensitivity of 63% and specificity of 91% for ultrasound combined with AFP for early-stage HCC detection, with significant heterogeneity of ultrasound performance, depending on the practice setting.^14^ Further ultrasound’s sensitivity for detecting early-stage HCC is lower in patients with obesity, alcohol-associated liver disease, and metabolic dysfunction–associated steatotic liver disease—increasingly common etiologies of cirrhosis in clinical practice.^15^^,^^16^ Up to 25% of patients undergoing HCC screening can experience screening-related harms due to indeterminate or false-positive abdominal ultrasound results.^17^^–^^19^ Finally, the effectiveness of ultrasound-based screening in clinical practice is limited by screening underuse due to patient and provider barriers.^20^^–^^24^ Overall, only 40% of patients with HCC are detected by screening, with most patients being detected incidentally or presenting symptomatically.^11^

Despite high sensitivity for early-stage HCC, imaging modalities, such as multiphase CT and contrast-enhanced MRI, are not recommended for routine HCC screening given possible harms (eg, radiation and contrast), lack of their cost-effectiveness, and limited radiologic capacity.^9^ There has been strong interest in adopting HCC screening strategies using emerging blood-based biomarkers since they are noninvasive, standardized, and easily applied in limited resource settings with potentially higher compliance rates.^25^ Therefore, biomarker-based strategies can improve screening effectiveness by increasing test sensitivity and adherence. One of the best-validated biomarker panels to date is GALAD, a 3-biomarker panel incorporating AFP, AFP-L3%, and des-carboxy prothrombin (all approved by the ultrasound Food and Drug Administration), combined with patient age and sex. In a multinational phase II biomarker study of 6834 patients (2430 patients with HCC and 4404 patients without HCC but with chronic liver disease), GALAD had AUROC >0.90 and sensitivities of 60%–80% for early HCC detection.^26^ GALAD has also been evaluated in 2 longitudinal retrospective cohort studies from the University of Michigan (n = 397) and Michael E. DeBakey VA Medical Center (n = 534).^27^^,^^28^ In the first study, 42 patients with HCC (57% early stage) were identified over a median of 2.0 years. At 90% specificity, the sensitivity of GALAD for any-stage and early-stage HCC were 72.0% and 53.8%, respectively. For the latter cohort, 50 HCC cases (68% early-stage) were identified over 1362 screening episodes. Using conventional cutoffs, GALAD had the highest sensitivity within 12 months of diagnosis, including 73.8% and 63.3% for all HCC and early-stage HCC, respectively.

GALAD recently completed evaluation in the Hepatocellular Carcinoma Early Detection Strategy (HEDS) phase III biomarker study, using a prospective collection retrospective blinded evaluation (ProBE) design.^29^ During a median follow-up of 40 months, 107 of 1550 patients with cirrhosis developed HCC (80% early stage). The AUROC for AFP and GALAD within 6 months of HCC diagnosis were 0.71 and 0.81, respectively. At a cutoff of −1.36, the sensitivity and specificity of GALAD within 6 months before HCC diagnosis were 65% (95% CI: 50%–78%) and 82% (80%–85%), respectively.^30^ Overall, these data underscore GALAD’s potential to improve early detection of preclinical HCC in patients with cirrhosis, warranting further validation.

The National Institutes of Health (NIH) Early Detection Research Network (EDRN) has proposed a standard pathway for the validation of cancer screening biomarkers.^31^ GALAD has been evaluated in phase II (case-control) and phase III (retrospective cohort) biomarker studies. Phase IV (prospective cohort) and phase V (RCT to evaluate screening-related mortality benefit) clinical utility studies are needed next to evaluate clinically important outcomes, including stage migration, curative-intent treatment receipt, HCC-related mortality, and screening-related harms from false-positive or indeterminate test results. Ultimately, these later-stage validation studies are required before the implementation of biomarker-based strategies in clinical practice. Herein, we describe the study protocol for the National Liver Cancer Screening Trial (TRACER), comparing GALAD and ultrasound in at-risk patients with cirrhosis from any etiology or those with chronic HBV infection.

### Objectives

The primary outcome of the trial is a reduction in late-stage HCC detection, defined as HCC beyond the Milan Criteria—the most common criteria used for liver transplantation in the West.^32^

Secondary outcomes of interest include: (1) proportion of patients detected beyond BCLC stage A, (2) screening utilization, (3) incidence of late-stage HCC, (4) curative therapy receipt, and (5) screening-related physical, financial, and psychological harms.

An exploratory outcome will be HCC-related mortality.

### Trial design

**FIGURE 1:**
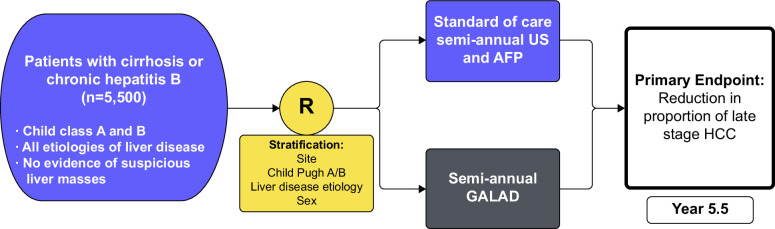
National Liver Cancer Screening Trial schema. Abbreviations: AFP, alpha-fetoprotein; US, ultrasound.

The TRACER Study is a pragmatic randomized phase IV trial comparing ultrasound-based screening versus the biomarker panel, GALAD, in patients with chronic HBV infection or cirrhosis from any etiology (Figure 1). In brief, 5500 patients are being randomized in a 1:1 fashion to arm A, recommending semi-annual screening with ultrasound ± AFP, or arm B, recommending semi-annual screening with GALAD. Randomization is stratified by site, Child-Pugh class (A vs. B), liver disease etiology (viral, nonviral, and noncirrhotic HBV infection), and sex. Compared to the EDRN-recommended pathway of a single-arm phase IV trial design, a randomized phase IV design for biomarker validation is advantageous in allowing a direct transition into a phase V study; this process would shorten the time to full validation if proven effective.

## METHODS

### Study setting

Patients are being recruited from 15 sites in the United States, including a mix of tertiary care academic referral centers, safety-net health systems, and large community health systems (Figure 1). Each site has a high volume of eligible patients (>1000 patients with cirrhosis each year), has expertise in enrolling patients with cirrhosis, and has capabilities to collect and store biorepository blood samples. Study sites were selected to ensure the geographic, racial, ethnic, and socioeconomic diversity of patients, given data demonstrating HCC disproportionately affects racial/ethnic minorities and underserved populations.^33^

### Eligibility criteria

Inclusion and exclusion criteria are listed in Table 1. The TRACER Study includes adult patients, age ≥18 years, with Child-Pugh class A or B cirrhosis of any etiology or noncirrhotic chronic HBV infection with PAGE-B score >9. The PAGE-B score was chosen to identify patients with HBV at the highest risk for incident HCC. PAGE-B has been extensively validated as an HCC risk-stratification score in both treatment-naïve and treatment-experienced patients with HBV.^34^ The presence of cirrhosis is defined based on histopathology, imaging showing a nodular, shrunken liver with signs of portal hypertension (eg, splenomegaly or intra-abdominal varices), or a noninvasive marker of fibrosis (eg, transient elastography, Fibrotest) consistent with cirrhosis.

**TABLE 1:** Inclusion and exclusion criteria

Inclusion criteria
Adult patients with cirrhosis of any etiology or noncirrhotic HBV infection with PAGE-B >9
Able and willing to provide informed consent
Child-Pugh A or B cirrhosis
Life expectancy >6 mo after consent
Exclusion criteria
Suspicious liver lesion (LR-3 ≥1 cm, LR-4, LR-5, and LR-M) within 6 mo
Lesion ≥1 cm on ultrasound or AFP ≥20 ng/mL within 6 mo, in the absence of CT or MRI showing lack of suspicious lesions
History of liver transplantation or listed for liver transplantation
History of any primary liver cancer
History of non-HCC malignancy within 3 y besides non-melanoma skin cancer or indolent tumor undergoing active surveillance
Child C cirrhosis
Undergoing routine CT or MRI screening
History of TIPS
History of cardiac cirrhosis
Known pregnancy
Active warfarin use

Abbreviations: AFP, alpha-fetoprotein; LR, Liver Imaging and Radiology Data System.

The TRACER Study excludes patients who are listed for or those who have undergone prior liver transplantation, patients with Child-ugh C cirrhosis, patients with significant comorbidity and limited life expectancy, and those with a history of other malignancy, except non-melanoma skin cancer or indolent tumors, within 3 years before enrollment given the lack of screening recommendations in those patient populations.^9^ Patients are also excluded if there are suspicious liver masses (Li-RADS [LR]-3 ≥1 cm, LR-4, or LR-M) within the 6 months before consent, given the high risk of prevalent HCC and need for CT or MRI-based surveillance of the nodules.^35^ The study also excludes those with a solid lesion ≥1 cm on ultrasound or AFP ≥20 ng/mL without diagnostic evaluation to exclude HCC. Patients with subcentimeter nodules on ultrasound are permitted given a low risk of HCC, permitting continued usual screening.^36^ Patients in whom their provider plans to follow the patient with CT or MRI-based screening are also excluded. Finally, GALAD is not recommended in patients with pregnancy or active warfarin use, given the known impact on biomarker performance (AFP and des-carboxy prothrombin, respectively), so these patients are excluded.

### Recruitment, patient consent, and retention

**FIGURE 2:**
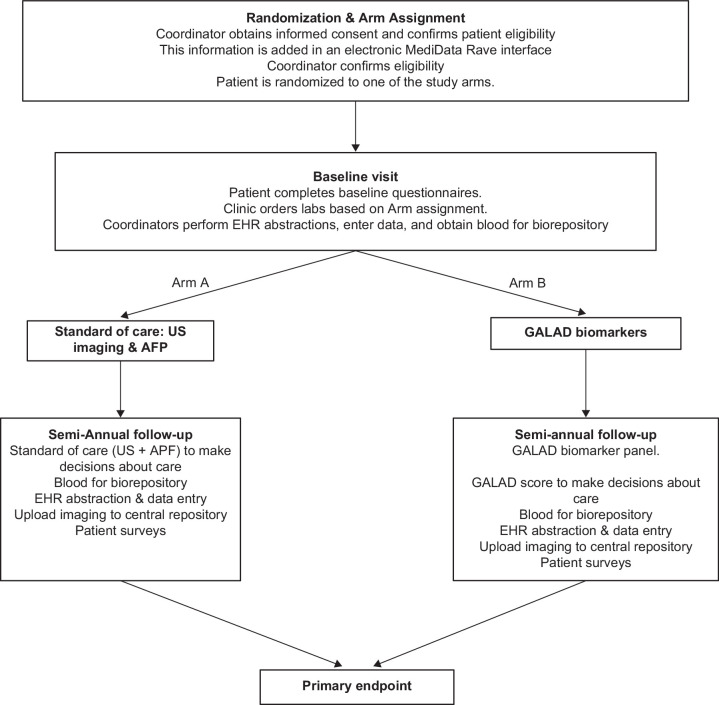
Participant timeline. Abbreviations: AFP, alpha-fetoprotein; EHR, electronic health record; US, ultrasound.

The participant timeline is detailed in Figure 2. Patients are being identified and enrolled from Hepatology clinics at the enrollment sites. During the clinic visit, the study coordinator confirms eligibility, administers informed consent, and collects relevant study data and blood specimens. This process allows study recruitment to be completed during routine clinic appointments, minimizes the burden on patients, and maximizes study recruitment. To ensure a diverse cohort, study materials, including consent forms, are translated into multiple languages, including Spanish, Mandarin, Vietnamese, and Korean. Patients are provided a $30 incentive at each visit to cover incidentals such as parking, with the goal of optimizing both study recruitment and retention.

## INTERVENTIONS

### Intervention description

Patients undergo semi-annual screening as defined by their study arm. For patients in arm A, semi-annual ultrasound ± AFP is recommended by clinical providers as per usual care, based on society guidelines; AFP is recommended but optional since there continues to be discordance across professional society recommendations about its use. For patients in arm B, semi-annual GALAD is recommended by the study team. When possible, GALAD is done at the time of collection of routine clinical labs to minimize patient burden. Whereas the recommendation for ultrasound ± AFP is deferred to usual care, the study team recommends GALAD in arm B to ensure consistency in ordering and calculating the panel. Therefore, the lack of an order for GALAD is a protocol deviation for arm B. To minimize differences in the intervention between the arms, the study team also sends a semi-annual notification to clinical providers of patients in arm A, reminding them that the patients are in the TRACER Study, with screening deferred to the standard of care. The study protocol does not include patient navigation in either arm, and patient nonadherence to screening is not a protocol deviation. Clinical providers of patients in both arms are discouraged from ordering contrast-enhanced abdominal CT or MRI for routine HCC screening, and providers of those in arm B are discouraged from ordering an abdominal ultrasound as routine HCC screening; however, these exams can be done for other purposes as clinically indicated (eg, imaging to evaluate abdominal pain) or if a clinical provider deems necessary.

Repeat screening tests are recommended every 6 months (per assigned arm) for patients with normal screening results (absence of liver lesion ≥1 cm on ultrasound and AFP <20 ng/mL for arm A; GALAD <−1.36 for arm B). For the GALAD arm, the study team notifies the clinic provider of positive GALAD screening results. Although multiphasic CT or contrast-enhanced MRI is recommended for any patient with abnormal screening results in both arms (ie, ultrasound liver lesion ≥1 cm, AFP >20 ng/mL, or GALAD ≥−1.36), diagnostic evaluation is at the discretion of patients’ clinic provider. Further, for both arms, the study does not include patient navigation (eg, reminder calls, scheduling assistance, and rescheduling for missed appointments) beyond what is available through routine clinical practice.

Patients with normal diagnostic testing (ie, false-positive screening result) are recommended to return to their assigned screening arm (ultrasound ± AFP for arm A and GALAD for arm B). Enrolled patients with incident indeterminate liver nodules (eg, LR-3, LR-4, or LR-M) are managed per the primary clinic provider. In brief, patients with LR-3 or LR-4 are typically followed by repeat CT/MR imaging, although biopsy may be indicated in some cases, particularly with larger lesions. Patients with LR-M are typically managed by biopsy, given the high risk of malignancy, including HCC. Although the study team does not recommend diagnostic testing for patients with indeterminate screening results (eg, subcentimeter lesions on ultrasound, suboptimal visualization on ultrasound, mild elevations of AFP or GALAD but not exceeding thresholds,^15^^,^^36^ elevated components of GALAD but normal composite score), this may be ordered by clinic providers as desired.

### Discontinuing or modifying the intervention

There are predefined cases in which patients would be removed from the study intervention (ie, recommending GALAD) but would continue routine clinical care such as clinic visits, labs, and imaging per their clinic provider:

1. patient voluntarily withdraws from GALAD-based screening;
2. patient is unable to comply with protocol requirements;
3. treating physician judges continuation on the study would not be in the patient’s best interest;
4. patient starts warfarin;
5. patient develops a malignancy (except for basal cell carcinoma or squamous cell carcinoma of the skin or indolent tumor that is undergoing active surveillance, eg, prostate cancer or renal cell carcinoma) that requires treatment, which would interfere with this study; and
6. patient is lost to follow-up.

If a patient becomes pregnant, GALAD would not be used until postpartum. All patients will be included in intention-to-screen analyses.

### Surveys and distribution

Study coordinators administer a set of validated surveys to measure secondary outcomes of interest including psychological and financial harms. Patients complete the following set of surveys at enrollment and annually during follow-up:

*Psychological Consequences Questionnaire* ^37^ is used to assess HCC-specific worry. As done for lung cancer screening, we use 3 questions: “Are you afraid you may have HCC?”; “Does the thought of HCC scare you?”; and “Are you afraid of dying soon from HCC?”

*Decision Regret Scale* ^38^ is a validated 5-item measure that assesses screening decisional conflict, satisfaction, and regret.

*FACIT*—*COmprehensive Score for Financial Toxicity* (*COST*)^39^ is an 11-item validated measure developed in patients with cancer to assess the psychological response to and the material condition of financial hardship.

*Out-of-pocket costs* includes direct (eg, copayment) and indirect (eg, parking, meals, childcare, and time-off-work) out-of-pocket cost data.

*Impact of Events Scale*,^40^ a validated 15-item questionnaire, is administered within 6 months after a positive screening result requiring a diagnostic evaluation to measure psychological distress. The survey was initially developed as a tool to evaluate posttraumatic stress disorder but has been modified to evaluate acute stress related to routine events, including the stress of positive screening results.

### Outcomes

The primary endpoint of the phase IV TRACER Study is the proportion of HCC detected at a late stage, as defined by the Milan criteria. Although there is no universally accepted staging system for HCC, 2 of the most common criteria for early-stage HCC in the Western world are the Milan Criteria (most common criteria for liver transplantation) and BCLC stage 0/A.^7^^,^^32^ One of the primary distinctions between the 2 is how large unifocal lesions are classified, with these being classified as early-stage HCC by the BCLC System but not the Milan Criteria. As these patients are typically not amenable to curative therapy in the Western world, the Milan Criteria were selected for the primary outcome.

Although the incidence of late-stage HCC is less prone to overdiagnosis and a preferred outcome for a phase IV study, it requires larger samples and/or longer follow-up beyond the available resources for current funding. Reduction in the proportion of late-stage cancer is a valid and acceptable outcome for phase IV cancer biomarker studies if overdiagnosis does not exist or the magnitude of overdiagnosis is small.^41^^,^^42^ HCC is traditionally considered an aggressive tumor with a low risk of overdiagnosis in patients with compensated liver disease and otherwise low comorbidity.^43^^,^^44^ Therefore, an adaptive trial design is used, whereby the primary outcome in the current funding cycle is the proportion of late-stage HCC, and the incidence of late-stage HCC is a secondary outcome; the trial then plans to continue follow-up to have adequate statistical power to evaluate the incidence of late-stage HCC in the future.

Secondary outcomes of interest include (1) the proportion of patients detected beyond BCLC stage A, (2) screening utilization, (3) incidence of late-stage HCC, (4) curative therapy receipt, and (5) screening-related physical, financial, and psychological harms. HCC-related mortality will be assessed as an exploratory outcome. *BCLC stage A* is defined as a single tumor of any size without vascular invasion or extrahepatic spread, or 2–3 tumors ≤3 cm each, without vascular invasion or extrahepatic spread, and includes Child-Pugh class and performance status. *Screening utilization* will be measured by the proportion of time covered (PTC) by screening.^45^ Each completed screening test (ultrasound/AFP or GALAD) provides up to 6 months of coverage; the number of months with screening coverage is divided by the total follow-up for each participant. Patients are considered to have 100% PTC if screening exams are 6 months apart or more frequent throughout follow-up, whereas any gap in screening completion exceeding 6 months results in reduced PTC. *Incidence of late-stage HCC* is often similar to the proportion of late-stage HCC, although the former can be a better surrogate of cancer-related mortality for cancers with a high risk of overdiagnosis (which is unlikely the case for HCC). *Curative therapy receipt* can be another surrogate for cancer-related mortality, given the stark difference in survival between patients who undergo curative versus palliative therapies. Early tumor detection without curative therapy may simply result in lead time bias rather than true change in prognosis. Conversely, patients who are detected at later stages who undergo curative therapy can achieve long-term survival. Curative therapies are defined as liver transplantation, surgical resection, or local ablative therapy. *Screening-related physical, financial, and psychological harms* will be assessed. Physical harms are defined as the receipt of diagnostic imaging or invasive testing for false-positive or indeterminate results. Direct financial harms are captured by charges for all screening and diagnostic testing. Co-pays and indirect costs (eg, travel and lost wages) are captured using surveys that are being conducted annually during the study period. Similarly, psychological harms, including cancer-specific worry and anxiety, are captured through annual surveys. *HCC-related mortality* will be defined as death from any liver-related cause in patients with HCC beyond an early stage. If the TRACER Study meets its primary outcome at year 5.5, follow-up will be extended to assess the incidence of late-stage HCC at year 8 and a decision will be made about adapting the current trial to a phase V design with sufficient power to assess reduction in HCC-related mortality.

## ASSIGNMENT OF INTERVENTIONS: ALLOCATION AND BLINDING

### Sequence generation

The data managing coordinating center (DMCC) of the EDRN generates the randomization sequence using a permuted block randomization scheme with varying block sizes (4, 6, and 8). Blocks are dynamically allocated to each stratum (determined by site, liver disease etiology, Child-Pugh class, and sex) during study enrollment.

### Concealment mechanism and implementation

Randomization is performed by the Medidata Rave system being used for central data management of the trial. All sites and investigators are blinded to the randomization sequence. During the enrollment clinic visit, once the patient has completed the informed consent process, study coordinators enter the necessary information into the Medidata Rave system, which then randomizes the patient and informs the study coordinator of the screening arm allocation.

## DATA COLLECTION AND MANAGEMENT

### Data collection

Patient demographics and clinical characteristics are being collected using a combination of electronic medical records and patient questionnaires and recorded into the Medidata Rave database. Demographics include age, sex, race, ethnicity, and socioeconomic status. Medical comorbidity with attention to metabolic syndrome components and alcohol history is being collected. The body mass index, which is measured using height and weight measurements, is recorded by study coordinators. Liver disease etiology is classified as viremic hepatitis C, post-SVR hepatitis C, chronic HBV, alcohol-associated, metabolic dysfunction–associated steatotic liver disease, or other etiology using data from the electronic medical record. Liver disease severity is assessed at enrollment, including laboratory values and the presence of gastro-esophageal varices, HE, and ascites. Gastro-esophageal varices are categorized as present versus absent, while ascites and encephalopathy are categorized as none, mild, or severe using standardized criteria. Laboratory data are recorded at enrollment, including white blood cell count, platelet count, sodium creatinine, bilirubin, albumin, and INR.

### Assessment of outcomes

Patients are followed every 6 months, with diagnostic evaluation performed in those with abnormal screening results. Standardized criteria from the AASLD and LI-RADS are used to define incident HCC, which include histological and/or radiological criteria based on a characteristic appearance (arterial enhancement and delayed washout) on multiphasic CT or contrast-enhanced MRI.^9^^,^^46^ Radiologic imaging (CT or MRI) with LR-3 ≥1 cm, LR-4 (“suspicious” for HCC), LR-5 (“definite” for HCC), LR-M (worrisome for malignancy, not definite HCC), and LR-TIV (suspected tumor in vein) are reviewed by a central adjudication committee consisting of 2 expert abdominal fellowship-trained radiologists. For all patients with confirmed HCC, the central adjudication committee also adjudicates tumor stage—both according to Milan Criteria and BCLC Staging System. Given HCC is typically diagnosed radiographically, most patients will not undergo biopsy. For those who undergo a biopsy showing suspected or definite liver cancer (HCC, cholangiocarcinoma, or mixed tumors), pathology reports and slides are reviewed by a central GI/liver pathology fellowship-trained pathologist to confirm the diagnosis.

### Collection and storage of biospecimens

Blood samples are being collected every 6 months to create a biorepository for validation of future biomarkers. A total of 30 mL of blood specimens (one 10 mL red top Vacutainer and two 10 mL purple top Vacutainers) are collected every 6 months. The biorepository collection is being done at the time of clinic or radiology visits, when possible, to minimize patient respondent burden. Specimens are processed and placed into 0.25–0.5 mL serum and plasma aliquots at the sites. Specimens are processed using protocols that are compatible for liquid biopsy biomarkers, including methylated DNA panels and extracellular vesicle-based analyses, including processing and freezing within 4 hours of collection (Supplemental Materials, http://links.lww.com/HC9/B73). Specimens are de-identified and labeled with barcodes that allow tracking of specimen creation, shipping, and receipt, as well as facilitate linkage to clinical data obtained for each participant. Specimens are being stored at the National Cancer Institute’s (NCI) Frederick after initial collection.

Contrast-enhanced MRI or multiphasic CT images for patients with incident HCC or any suspicious liver lesions (LR-4, LR-M, or LR-TIV) are also being de-identified and stored. After central adjudication, images will be transferred to the NIH central biorepository for future analyses. Exam indication is recorded from clinical notes and the imaging report and classified as screening, diagnostic for abnormal screening results, or diagnostic for other indications. Doing so also allows blinded analyses for any imaging-based biomarkers, including automated and quantitative radiomics detection and characterization.

## STATISTICAL METHODS

### Statistical methods for primary and secondary outcomes

The primary endpoint that will be assessed at year 5.5 is the proportion of HCC cases that are diagnosed at a late stage, defined as HCC beyond the early stage per Milan Criteria. The proportion of late-stage HCC will be compared across the 2 screening arms. Currently, there is no evidence in the literature to indicate that overdiagnosis is a concern in HCC; however we have chosen to be conservative and conduct our primary analysis with a correction for 5% overdiagnosis in the GALAD arm using the method of Chari et al.^41^

Specifically, the primary evaluation of intervention effect using test statistic:


$$T=n_{A,L-\mathrm{HCC}}/n_{A,\mathrm{HCC}}–n_{B,L-\mathrm{HCC}}/\left[n_{B,\mathrm{HCC}}/\left(1+f\right)\right]$$

where *n*_A,L-HCC_ is the number of observed late-stage HCC cases in arm A (ultrasound ± AFP), *n*_A,HCC_ is the number of observed HCC cases in arm A, *n*_B,L-HCC_ is the number of observed later-stage HCC cases in arm B (GALAD), *n*_B,HCC_ is the number of observed HCC cases in arm B, and *f* is the assumed upper bound of overdiagnosis. We decided to use *f* = 0.05. We will conduct a one-sided test for the superiority of GALAD arm versus the ultrasound ± AFP arm at the significance level of 5%.

The evaluable population for the primary analysis will be intention-to-screen, including all patients who meet eligibility criteria and are enrolled in the study and randomized.

A secondary endpoint will be to describe screening utilization of recommending ultrasound ± AFP versus GALAD screening. Screening utilization will be defined as the PTC by screening. Each completed screening test (ultrasound ± AFP or GALAD) will provide up to 6 months of coverage (numerator) divided by the total follow-up for each patient. We will report the weighted mean PTC in each arm (where patients are weighted by the total time in follow-up) since patients with longer follow-ups should contribute more information.

In secondary efficacy analyses, we will compare the proportion of HCC detected at a late stage defined to be BCLC stage B or higher using the same method described for our primary analysis with a correction for 5% overdiagnosis in the GALAD arm. The incidence of late-stage HCC (defined using both the Milan Criteria and BCLC criterion) will be compared between the 2 arms using a one-sided Poisson test for the superiority of the GALAD arm versus ultrasound ± AFP arm at the significance level of 5%. At the end of year 5.5, we expect to be underpowered (~60% power) to detect a reduction in the risk of late-stage HCC. At year 8, we expect to have 83% power to detect a 39% reduction in the incidence of late-stage HCC for the GALAD arm (0.43 late-stage HCC per 100-patient year) compared to the ultrasound ± AFP arm (0.71 late-stage HCC per 100-patient year). The proportion of HCC cases that receive curative therapy, defined to be receipt of liver transplantation, surgical resection, local ablative therapy, or radiation segmentectomy, will be compared across the 2 arms using a one-sided test for proportions at the significance level of 5%.

A secondary objective of the study will be to evaluate the safety of recommending screening, as measured by screening-related physical, financial, and psychological harms. Physical harms will be defined as the receipt of diagnostic imaging for false-positive results. The total number of occasions where patients receive diagnostic imaging for false-positive results (defined as a screening test positive but no HCC diagnosed within 12 mo) will be evaluated within each arm. Financial harms will be defined by direct costs (charges for all screening and diagnostic testing and co-pays) and indirect costs (eg, travel and lost wages). These will be assessed using the out-of-pocket cost survey that will be conducted annually. These costs will be described over the course of the study within each arm by summarizing average costs over time. Financial harms will also be assessed using the FACIT survey that will also be conducted annually. The FACIT COST score will be obtained using the scoring guidelines provided, where higher scores indicate better financial well-being. The FACIT COST score will be described over the course of the study within each arm by summarizing the average score over time.

Psychological harms will include cancer-specific worry and decisional regret. To assess HCC-specific worry, adapted questions from the Psychological Consequences Questionnaire are assessed annually, and the changes in responses over time will be described within each arm. The Decision Regret Scale will be assessed using the scoring guidelines provided, where higher scores mean higher regret. The Decision Regret Scale score will be described over the course of the study within each arm by summarizing the average score over time. Finally, we will use the Impact of Event Scale in patients with positive screening results to measure psychological distress due to true-positive or false-positive results.

### Sample size calculation

The study will enroll 5500 patients during the first 3 years, and the primary analysis will be conducted 5.5 years after study enrollment begins when the average follow-up for each patient is 2.6 years. Assuming an annual HCC incidence of 2%, we anticipate a total of 303 HCC cases over 14,500 person-years of follow-up. The expected proportion of late-stage HCC in the SOC arm is 39%. Therefore, the study will have 81% power to reject the null hypothesis using a one-sided test at the 5% significance level if the observed proportion of late-stage HCC in the GALAD arm is 24%, which corresponds to a 38% reduction in the proportion of late-stage HCC, assuming there is 5% overdiagnosis in the GALAD arm.

The sample size was determined using a Monte Carlo simulation study (with 10,000 trials) to model the effect of screening interventions in patients with cirrhosis. The first step was to simulate outcomes for patients with cirrhosis in the absence of HCC screening. We then implemented screening with either ultrasound ± AFP or GALAD. The sensitivity, specificity, and adherence of each screening approach were our key assumptions. The simulation assumed that in patients who would be diagnosed with late-stage HCC in the absence of any screening, screening with either ultrasound ± AFP or GALAD may result in a stage shift, depending on test performance and adherence to screening. A patient would experience a stage shift because of screening testing if they had a positive screening test in the window 6–18 months before their late-stage HCC clinical diagnosis. Therefore, both the assumed sensitivity of the test and the adherence assumption would affect if a late-stage HCC case experiences a stage shift to early-stage HCC, and the proportion of late-stage HCC is a consequence of these assumptions.

We assumed 60% sensitivity, 85% specificity, and 55% adherence in the ultrasound ± AFP arm based on a study that found ultrasound with AFP increases early-stage sensitivity to 63%, but this is offset by a decrease in specificity to 84%.^14^ In a study that examined receipt of screening in 629 patients diagnosed with HCC, 14% of patients received semi-annual screening, and 22% of patients received annual screening, while ~64% received less than annual screening.^21^ We have chosen to be more conservative and used a higher adherence assumption of 55% (rather than 36%) in the ultrasound ± AFP arm since these patients are enrolled in a trial, and this can affect the behavior of both providers and patients. The same study found that the proportion of late-stage HCC under the current standard of care is ~35%. Note that the 5 sites where this was studied are part of this clinical utility trial. Given that we are enrolling more community-based sites in this clinical utility trial where the proportion of late-stage HCC can be as high as 60%,^47^ we believe 39% late-stage HCC in the ultrasound ± AFP arm is justified.

We assumed a sensitivity of 70%, specificity of 80%, and adherence of 80% in the GALAD arm. The sensitivity assumptions are based on results from the HEDS cohort, which found that at a cutoff of −1.36, sensitivity and specificity of GALAD within 6 months before HCC diagnosis were 65% (95% CI: 52%–78%) and 82% (80%–84%), respectively and THCCC cohorts where the sensitivity within 6 months before the diagnosis of early-stage HCC was 73% and specificity was 74%.^29^ Note that the specificity assumption does not affect our primary endpoint of the proportion of late-stage HCC but could affect secondary endpoints about the consequences of false-positive screening tests. Adherence of 80% is justified by further analysis in the HEDS cohort, where, on average, patients completed labs at ~65% of all study visits and ~83% of study visits with some record of study contact. Therefore, we believe achieving 80% adherence in the GALAD arm is a justifiable assumption given that the trial includes the research team ordering the GALAD testing, and GALAD can be completed at local labs (ie, does not require a visit to the health system main campus).

Enrollment is expected to be completed over 3 years. The study initially launched with 10 sites, and we assume that each site requires 6 months to ramp up enrollment from 7 patients/month to the target enrollment rate of 12 patients/mo. As of September 2024, an additional 5 sites are being launched. These sites are expected to require 6 months to reach target enrollment of 12 patients/mo. Therefore, the accrual of 5500 patients into the trial will require 35 months of enrollment. The power calculations consider multiple reasons why patients may not have an observed HCC event. We assume ~10% lost to follow-up by the end of 5.5 years, and we have also accounted for the competing risk of death from other causes, removal due to transplantation, and sporadic missed visits during the study. Note that our study will aim to continue to follow-up patients for outcomes of tumor stage and survival among patients who withdraw from the study intervention by asking them to consent to medical record review. Therefore, the estimate of 10.6% refers to patients who are alive but stop all medical visits at some point during the study, and, therefore, we believe it is a reasonable assumption that yields 81% power with our current design of 5500 patients. Our power estimates are robust to loss to follow-up. The power decreases to 80.4%, with 15% of patients lost to follow-up and 80% with 20% loss to follow-up. The reason that loss to follow-up has minimal effect on the study power is because the larger source of censoring for our primary endpoint of HCC events is death due to other causes or liver transplantation. We assume removal from the cohort for death or transplantation due to cirrhosis is based on Child-Pugh status at entry (2-y survival with Child-Pugh score A is 90%, 2-y survival with Child-Pugh score B is 60%, the probability of being Child-Pugh A at entry is assumed to be 60%). By the end of the study period, the average probability of being lost to follow-up is 11%, and the average probability of death or transplant before HCC is 45%.

Therefore, we believe that with 5500 patients followed for an average of 2.6 years, we will observe a clinically significant reduction in the proportion of late-stage HCC.

### Subgroup analyses

The proportion of late-stage HCC will be compared across the 2 arms among patients with documented compliance with the assigned screening strategy. A patient in the GALAD screening arm will be considered compliant with the assigned screening strategy if they complete testing for at least 80% of the study windows and have <15% of study windows with screening imaging performed. A patient in the ultrasound ± AFP screening arm will be considered compliant with the assigned screening strategy if they undergo ultrasound ± AFP testing for at least 80% of the study windows and have <15% of study windows with screening CT/MRI performed.

### Procedures to address nonadherence and missing data

Our sample size calculation assumes some level of nonadherence, specifically contamination with MRI/CT screening, since we used estimates from the HEDS and THCCC studies where this contamination was present. However, the study includes regular reminders to providers to avoid using MRI/CT for screening in patients enrolled in the study, and the rates of contamination and nonadherence are being monitored during the trial conduct. Missing data are minimized in the study since most data can be abstracted from the medical record, but our sample size calculations indicate that we are protected in our study power for up to 20% loss to follow-up. Patients who are lost to follow-up are censored at the last known time they were alive and cancer-free. For our primary analysis, missing data for HCC stage are minimized through central review; however, if a patient is not able to be staged, we will assume they have late-stage HCC. We will conduct a sensitivity analysis in which patients who have missing stage are removed. For secondary endpoints, missing data will be clearly described and included in the reporting of trial results.

### Access to participant-level data or specimens

Data that support the findings of this study will belong to a biorepository intended for continued blinded validation of biomarkers. Access to samples or data after study completion is possible through an application process, which undergoes scientific review by members of the EDRN Biorepository Committee and Liver Cancer Clinical Validation Center. For information regarding the application process for specimens and data, please see https://edrn.nci.nih.gov/data-and-resources/specimen-reference-sets.

## OVERSIGHT MONITORING

### Data monitoring committee and reporting structure

A data safety monitoring board (DSMB) has been established to ensure human subjects’ protection, proper conduct of the trial, and integrity of the study data. The DSMB evaluates trial progress, including participant recruitment and retention rates, performance of trial sites, participant risk versus benefit ratio, and any other factors that can affect study outcome. In addition to reviewing adverse events (AEs), the DSMB assesses for futility/harm at predefined time points using criteria defined in conjunction with the DMCC. At each DSMB meeting, the DMCC provides an estimate of the posterior probability of harm based on a Bayesian logistic regression modeling approach. In this setting, harm refers to observing a higher probability of late-stage HCC in the GALAD arm compared to the ultrasound ± AFP arm. The hyperparameters of the prior distributions were selected to reflect the expected proportion of late-stage HCC in the ultrasound ± AFP arm and a prior distribution of harm with a mean of 0. There are no prespecified boundaries, but rather this information is provided to the DSMB to make informed recommendations based on benefit, harm, strength of the data, and uncertainty trade-offs. After each meeting, the DSMB makes a recommendation to continue accrual without protocol changes, continue accrual with protocol changes, suspend accrual pending protocol changes, or close the study.

### Adverse event reporting and harms

The TRACER Study involves the administration of questionnaires, data collection, imaging, and blood collection. The likelihood of serious AEs during participation in this protocol is low, although serious AEs directly related to study data collection or blood draw will be reported. Lack of screening and occurrence of late-stage HCC are not reportable AEs; however, the differences in early versus late-stage HCC between the 2 arms will be monitored by the DMCC. Screening tests and diagnostic procedures can promote downstream complications, and AEs associated with these diagnostic procedures are termed indirect AEs.

## DISCUSSION

In summary, HCC remains one of the few cancers with a 5-year survival below 20% and rising mortality in the West, largely related to frequent late-stage diagnosis. Ultrasound-based screening, the current standard of care, has suboptimal sensitivity for early-stage HCC and is frequently underused in clinical practice.^14^^,^^20^ These limitations underscore the importance of novel screening strategies to improve early-stage HCC detection. Although there are several emerging blood- and imaging-based screening strategies, GALAD is the only one to complete evaluation in a phase 3 biomarker study.^25^^,^^29^ The TRACER study is a phase 4 biomarker study that directly compares GALAD, a blood-based biomarker panel, versus ultrasound for early-stage HCC detection.

Phase 2 (case-control) and phase 3 (retrospective cohort) biomarker studies provide estimates of test performance, that is, sensitivity and specificity, whereas phase 4 and phase 5 (prospective cohort and RCT studies) are important to assess clinical utility.^48^ Although prior studies have shown that GALAD has sufficient sensitivity and specificity compared to ultrasound, TRACER will be critical to understanding its ability to improve clinical outcomes, including early-stage HCC detection, curative treatment receipt, and, most notably, HCC-related mortality. Further, TRACER will provide important insights into potential screening-related physical, financial, and psychological harms.^17^^–^^19^ Together, these data can help compare the overall value of the 2 strategies in at-risk patients. If GALAD demonstrates superiority to ultrasound, HCC screening will shift from an imaging-based to a biomarker-based strategy in clinical practice. Doing so may not only improve test sensitivity but also reduce patient barriers to HCC screening and thereby improve utilization and overall effectiveness of screening. If GALAD reduces late-stage HCC but the difference does not reach statistical significance, the interpretation of results will likely depend on comparisons in test performance and utilization. If GALAD achieved equal or higher sensitivity for early-stage HCC, then it is possible that the 2 strategies are noninferior (understanding the trial is not powered for noninferiority) and GALAD may be a viable alternative, particularly in populations with barriers to ultrasound-based screening.

Traditionally, biomarkers undergo single-arm prospective cohort validation examining early-stage detection before their evaluation in an RCT, which examines downstream outcomes, including cancer-related mortality. TRACER represents a methodologic innovation; conducting a randomized phase IV design (vs. traditional single-arm design) offers an opportunity to directly transition into phase V and thus shorten the time to full validation. These efforts are important given the historical long time from initial evaluation to adoption in clinical practice^6^ and can serve as a model to expedite the evaluation of emerging screening modalities in other cancers. If TRACER demonstrates a significant difference in the incidence of late-stage HCC and curative therapy receipt, it could be argued that further follow-up for HCC-related mortality may not be needed. As with many cancers, tumor stage and curative therapy receipts are widely recognized surrogates for overall survival in patients with HCC.

We have implemented strategies to address potential limitations. First, although both arms recommend the respective screening tests, ultrasound ± AFP and GALAD, testing is deferred to clinical providers in Arm A, and the absence of an order for ultrasound does not qualify as a protocol deviation. For patients in arm B, GALAD is ordered by the study team to ensure consistency in ordering and calculating the panel, so the lack of orders for GALAD is a protocol deviation. We acknowledge this could lead to enhanced screening promotion in arm B compared to arm A. To mitigate this potential issue, the study team sends a message to clinical providers about their patients who are enrolled in arm A, reminding them that their screening should proceed as per the standard of care. This ensures a similar frequency of contact with clinical providers in both arms. We do not offer any additional navigation, and failure to adhere to recommended screening tests is not a protocol deviation in this pragmatic trial. Second, our power calculations assume an annual HCC incidence of 2%, although the event rate may be lower considering the epidemiologic shift in HCC risk factors to nonviral etiologies; however, recent studies have demonstrated incidence rates of 1.5%–2% per year in contemporary cohorts.^4^ Third, there is a risk of ascertainment bias, given the lack of cross-sectional imaging in both arms. Although advanced-stage HCC cases should present clinically with continued follow-up, there may be a differential impact of ascertainment bias at the time of our analysis at 5.5 years. There is also a risk of overdiagnosis, where we detect cancer in patients with high competing risks of mortality or indolent tumors, given the inclusion of patients with Child-Pugh B cirrhosis as well as a reliance on imaging-based diagnosis; however, we incorporated this possibility into our sample size calculations.^43^ It is possible that overdiagnosis could differentially impact the 2 arms if one strategy is more prone to detecting indolent tumors, underscoring the importance of continued follow-up to evaluate HCC-related mortality.^44^^,^^49^

Finally, it is possible that emerging imaging and blood-based technologies, including abbreviated MRI and liquid biopsy biomarkers, may have better efficacy than GALAD.^50^^–^^54^ These strategies have completed phase 2 evaluation, but further validation of these strategies has been historically slower given a lack of clinically annotated biorepositories allowing phase 3 biomarker validation. For example, HEDS and Texas HCC Consortium have stored serum and plasma, but blood processing protocols were not consistent with liquid biopsy techniques, and there are not any centrally stored images. TRACER is creating an imaging biorepository and using a contemporary blood processing protocol to allow validation of liquid biopsy techniques. Doing so in parallel will also create one of the first large, diverse cohorts in which both are available, allowing the evaluation of imaging and biomarker combinations for early-stage HCC detection and diagnosis.

In summary, the TRACER Study is an innovative adaptive randomized clinical trial, comparing biomarker versus imaging-based screening, whose results will help inform the best strategy for HCC screening in patients with chronic liver diseases.

## Supplementary Material
