# Supplementary material for: National Liver Cancer Screening Trial (TRACER) study protocol
Source: Hepatol Commun. 2024 Nov 4;8(11):e0565. doi: 10.1097/HC9.0000000000000565 (PMC11537583; doi:10.1097/HC9.0000000000000565)
Supplement: Supplementary file 1 [file hc9-8-e0565-s001.docx]

# **Serum Preparation**

1. Blood will be collected in vacutainers with no anticoagulant (BD) in the form of red top tubes.
2. Each sample must be processed and stored within 4 hours of collection
3. After collection of the whole blood, allow the blood to clot by leaving it undisturbed standing upright at room temperature. This usually takes at least 30 minutes, but not more than 60 minutes.
4. After allowing the sample to clot, place the tube(s) in the refrigerator at 4°C until processing.
5. Spin for 20min at 1300g, must use refrigerated centrifuge (4°C)
6. The samples should be maintained at 2-8°C using ice bucket while handling, *as needed*
7. Aliquot into cryovials, and store all aliquots at –80°C

# **Plasma Preparation**

1. Collect whole blood into purple top vacutainer with EDTA
2. Each sample must be processed and stored within 4 hours of collection
3. Invert 8-10 times, store upright in 4°C refrigerator until processing
4. Spin for 10min at 530g with break ON 9/9, must use refrigerated centrifuge (4°C).
5. Immediately transfer the plasma into single new labeled 15-ml conical, avoid aspirating the buffy coat layer and the RBCs.
6. Spin for 10min at 4500g with break ON 9/9, must use refrigerated centrifuge (4°C).
7. The samples should be maintained at 2-8°C using ice bucket while handling, *as needed*
8. Aliquot into cryovials, and store all aliquots at –80°C
